# Hip Arthroscopy–Assisted Management of Pipkin Types I and II Femoral Head Fracture–Dislocations: Mid‐Term Clinical and Radiographic Outcomes

**DOI:** 10.1111/os.70387

**Published:** 2026-08-17

**Authors:** Shan‐Ling Hsu, Chi‐Hsiang Hsu, Chin‐Yi Liao

**Affiliations:** ^1^ The Department of Orthopaedic Surgery Kaohsiung Chang Gung Memorial Hospital and Chang Gung University College of Medicine Kaohsiung Taiwan; ^2^ Department of Orthopaedic Surgery Kaohsiung Municipal Fong Shan Hospital–Under the Management of Chang Gung Medical Foundation Kaohsiung Taiwan

**Keywords:** femoral head fracture, hip arthroscopy, Pipkin, trauma

## Abstract

**Objectives:**

Hip arthroscopy–assisted surgery has been proposed as a minimally invasive option for femoral head fractures; however, evidence with mid‐term follow‐up remains limited. This study aimed to evaluate the clinical and radiographic outcomes of arthroscopy‐assisted management for Pipkin Types I and II femoral head fracture–dislocations with a minimum follow‐up of 5 years.

**Methods:**

This retrospective study included 23 consecutive adults (19 Pipkin I and 4 Pipkin II) treated with hip arthroscopy–assisted fragment excision or internal fixation between March 2013 and January 2020. Preoperative computed tomography was used for surgical planning, and fixation was placed with arthroscopic headless screws. Clinical outcomes were assessed using the Harris Hip Score (HHS) and Thompson–Epstein (T–E) criteria. Radiographic evaluation included avascular necrosis (AVN), heterotopic ossification (HO; Brooker), osteoarthritis (OA; Tönnis), and fracture reduction quality (Matta's criteria). Group comparisons were evaluated using independent samples *t*‐tests, Mann–Whitney *U* tests, and Fisher's exact test. The mean follow‐up was 86.2 ± 21.2 months.

**Results:**

The cohort consisted of 19 males and 4 females with a mean age of 28.7 ± 9.9 years. Fifteen patients underwent fixation and eight underwent excision. The final mean HHS was 98.3 ± 1.9, with 21 patients (91%) achieving excellent and 2 (9%) good T–E criteria. There were no significant differences between the fixation and excision groups in demographic characteristics, operative time, or functional outcomes (all *p* > 0.05); however, hospital stay was significantly shorter in the excision group (2.9 ± 0.6 vs. 5.5 ± 4.6 days, *p* = 0.028). In the fixation group, mean maximal displacement improved from 7.6 mm preoperatively to 2.6 mm postoperatively, with anatomic reduction achieved in 6 cases (40%), imperfect in 6 (40%), and poor in 3 (20%). Patients with Pipkin Type I fractures had significantly higher HHS than those with Type II fractures (98.7 ± 1.7 vs. 96.0 ± 0.8, *p* = 0.018). Complications were rare, with one case of Brooker Grade I HO and one case of mild OA. No AVN or total hip arthroplasty occurred during the follow‐up.

**Conclusions:**

Hip arthroscopy–assisted management of selected Pipkin Type I and II femoral head fractures yields excellent mid‐term clinical outcomes with acceptable radiographic reduction and a low complication rate. This minimally invasive technique represents a viable alternative in appropriately selected patients when fragment characteristics and surgical expertise permit.

## Graphical abstract

This study showed that arthroscopic fixation for femoral head fractures yields excellent functional outcomes and low complication rates in the mid‐term follow‐up.

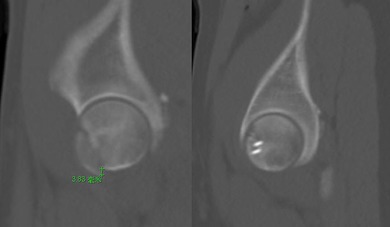

**Abbreviations**
3D‐CT: computed tomography with three‐dimensional reconstruction
AVN: avascular necrosis
HHS: Harris Hip Score
HO: heterotopic ossification
OA: osteoarthritis
POD1: Postoperative Day 1
T–E: Thompson and Epstein
VAS: Visual Analog Scale

## 1 Introduction

Fractures of the femoral head are rare but devastating injuries, accounting for approximately 6%–15% of traumatic posterior hip dislocations. These injuries typically result from high‐energy trauma, such as motor vehicle accidents or falls from height, in which axial force is transmitted through the femur while the hip is flexed. The Pipkin classification remains the most widely utilized system, stratifying these fractures based on the fragment's relationship to the fovea capitis and the presence of associated femoral neck or acetabular fractures.

The optimal management of Pipkin Type I and II fractures remains a subject of significant debate in orthopedic trauma. While conservative treatment often leads to joint incongruity and loose bodies, surgical intervention is generally indicated for displaced fragments, mechanical blocks to motion, or joint instability [1, 2, 3]. Traditionally, open reduction and internal fixation via the Smith–Petersen, Kocher–Langenbeck, or surgical hip dislocation approaches have been regarded as the standard. While open techniques allow direct visualization and manipulation of fracture fragments, they are often associated with extensive soft‐tissue dissection, substantial blood loss, and a high incidence of heterotopic ossification (HO). Moreover, avascular necrosis (AVN) of the femoral head remains a major concern, as preservation of the femoral head's tenuous blood supply is critical for long‐term joint survival [4, 5, 6].

Recently, hip arthroscopy has emerged as a minimally invasive alternative for the management of selected femoral head fractures. Although arthroscopy is technically demanding and visualization may be limited, particularly in the medial compartment, it provides a magnified, high‐definition intra‐articular view of the weight‐bearing femoral head surface and cotyloid fossa—regions that often require extensive exposure during open surgery. Arthroscopy facilitates targeted removal of loose bodies, treatment of concomitant labral pathology, and fragment fixation or excision while minimizing soft‐tissue disruption. Despite these theoretical advantages, existing evidence is largely limited to small case series with short‐term follow‐up, and mid‐term outcomes—particularly regarding joint preservation and functional recovery—remain insufficiently reported [7, 8].

Therefore, the purpose of this study was to evaluate the clinical and radiographic outcomes of arthroscopy‐assisted surgery for Pipkin Type I and II fractures with a minimum follow‐up of 5 years. We specifically aimed to (i) compare functional outcomes and complication rates between fragment fixation and excision, (ii) compare results between Pipkin Type I and II fractures, and (iii) assess whether the fracture reduction quality influences mid‐term outcomes.

## 2 Methods

### 2.1 Study Design

This retrospective consecutive case series was conducted at a single tertiary referral center. The inclusion criteria were (i) displaced femoral head fracture–dislocations, and (ii) initial treatment with hip arthroscopy–assisted surgery between March 2013 and January 2020. The exclusion criteria were (i) Pipkin Type IV fractures, (ii) femoral head impaction (Chiron Type V) fractures, and (iii) the need for intraoperative conversion to open surgery.

**FIGURE 1:**
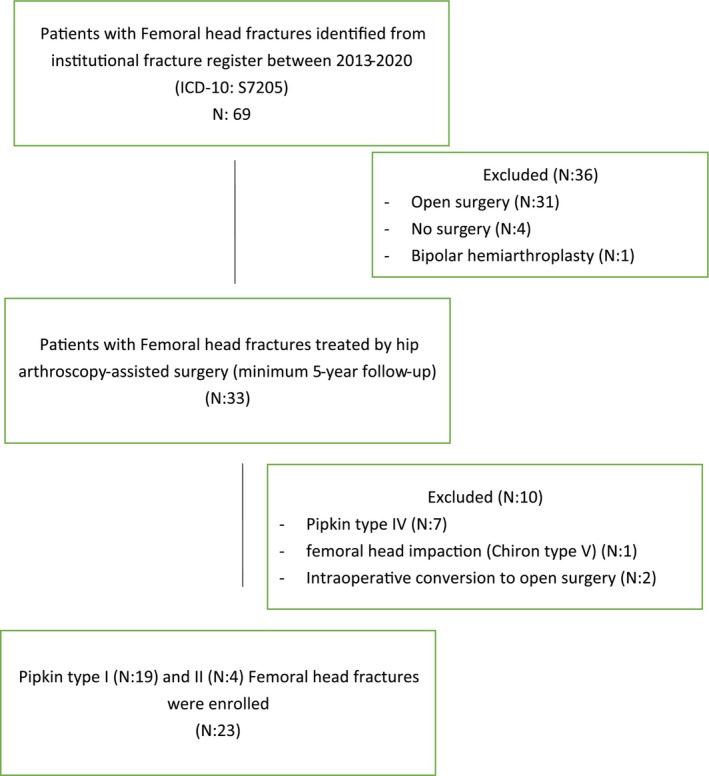
Flowchart of patients' included and excluded criteria in the study.

Based on these criteria, 69 patients were initially identified from the institutional trauma database. Following emergency closed reduction, all patients underwent standardized radiographic evaluation, including plain radiographs and computed tomography with three‐dimensional reconstruction (3D‐CT). The final cohort comprised 23 patients (Figure 1). Written informed consent was obtained from all patients before surgery. Demographic data and perioperative variables, including age at surgery, sex, Visual Analog Scale (VAS) pain score, intraoperative blood loss, operative time, length of hospital stay, and postoperative complications, were recorded from medical records.

### 2.2 Surgical Indications

The surgical indications for Pipkin Type I fractures were standardized based on preoperative 3D‐CT imaging and intraoperative findings:

- *Internal fixation*: Reserved for Type II fractures and Type I fractures with fragments that were large (≥ 2 cm in maximum diameter), displaced by > 2 mm, and deemed reducible to maintain joint congruity.
- *Fragment excision*: Performed for Type I fractures where fragments were small (< 2 cm), comminuted, or irreducible, particularly when they did not involve the primary weight‐bearing surface.

This selective approach ensures that potentially obstructive fragments are removed to prevent cartilage irritation, while significant structural fragments are stabilized to preserve the integrity of the femoral head [9].

### 2.3 Surgical Methods

All procedures were performed under general anesthesia with the patient in the supine position on a fracture table with intermittent traction applied to facilitate joint distraction.

#### 2.3.1 Portal Placement and Joint Visualization

Three standard arthroscopic portals were utilized to visualize the hip joint using a 70° arthroscope. To ensure safe portal placement and avoid vascular injury, the location of the femoral artery was identified using ultrasonography before the procedure.

#### 2.3.2 Reduction Technique

The hip joint was positioned in maximal external rotation to bring the fracture fragment into the superior operative field for optimal visualization and manipulation. Fracture reduction was performed using a joystick technique, in which instruments such as a surgical grasper, elevator, or probe were used to manipulate the fragment into an anatomic or near‐anatomic position under arthroscopic visualization.

#### 2.3.3 Fixation and Verification

**FIGURE 2:**
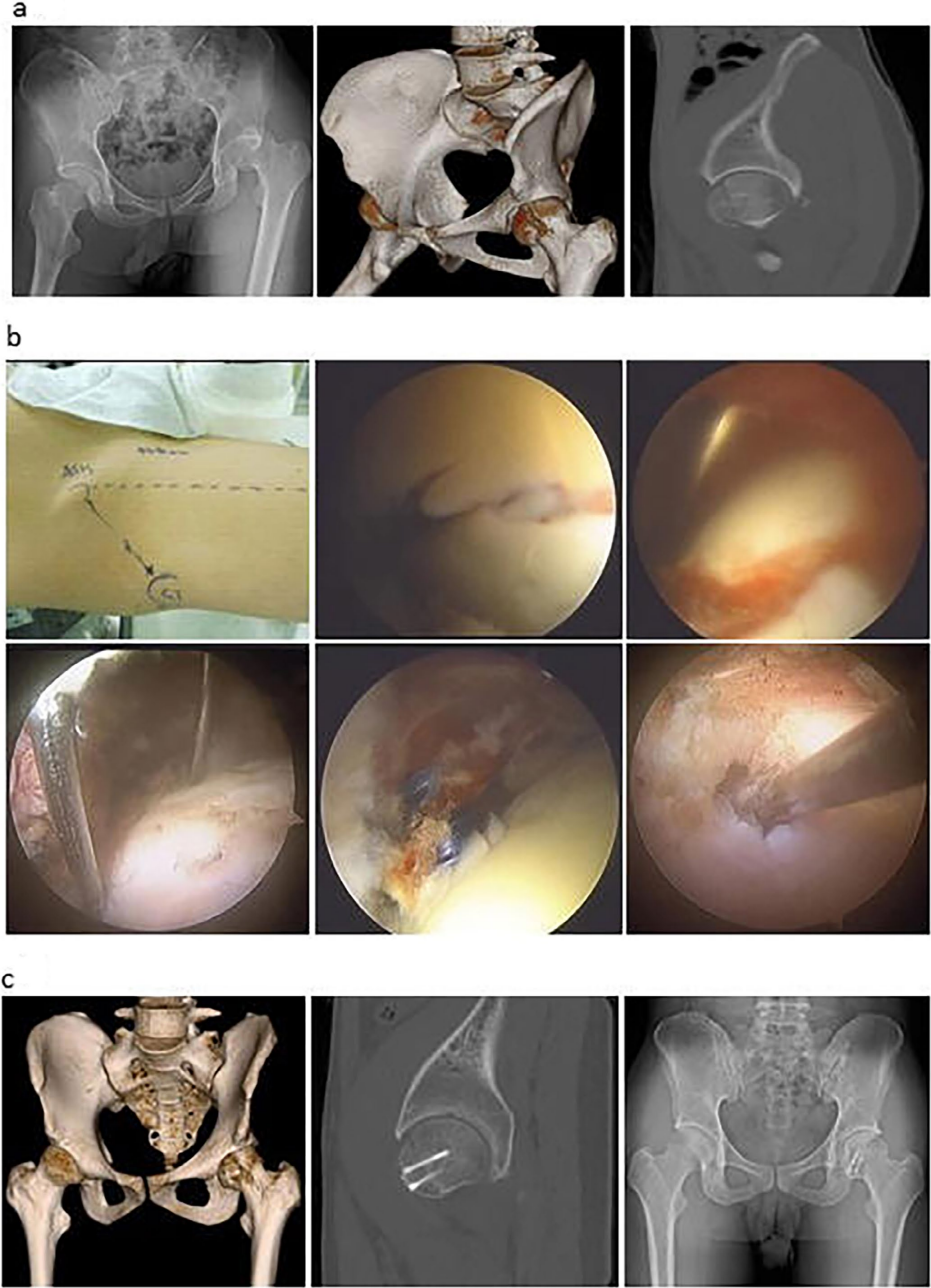
A 21‐year‐old female with a left Pipkin Type I fracture. (a) Preoperative imaging; (b) intraoperative arthroscopy–assisted reduction and fixation technique. Preoperative surface marking and identification of the femoral artery using ultrasonography for safe portal placement. Arthroscopic view from the anterolateral portal showing the displaced femoral head fragment. Use of the “joystick technique” with a probe and elevator to manipulate the fragment. Achievement of fracture reduction under direct visualization. Percutaneous insertion of guide wires and definitive fixation with headless compression screws. Final arthroscopic inspection confirming that screws are properly countersunk beneath the subchondral bone with no intra‐articular protrusion; (c) 6‐year follow‐up showing fracture healing and joint congruency without screw migration.

After satisfactory reduction was achieved, percutaneous guide wires were inserted through blunt muscle dissection under fluoroscopic guidance. The entry points were located between the anterior arthroscopic portal and the anterior superior iliac spine (ASIS). Under combined fluoroscopic and arthroscopic guidance, the guide wires were advanced toward the fracture fragment, with the tips positioned as close as possible to the center of the fragment. Definitive fixation was then performed using two or three 2.0‐mm Herbert screws, which were countersunk beneath the subchondral bone to prevent protrusion into the articular surface (Figure 2).

#### 2.3.4 Quality Control

**FIGURE 3:**
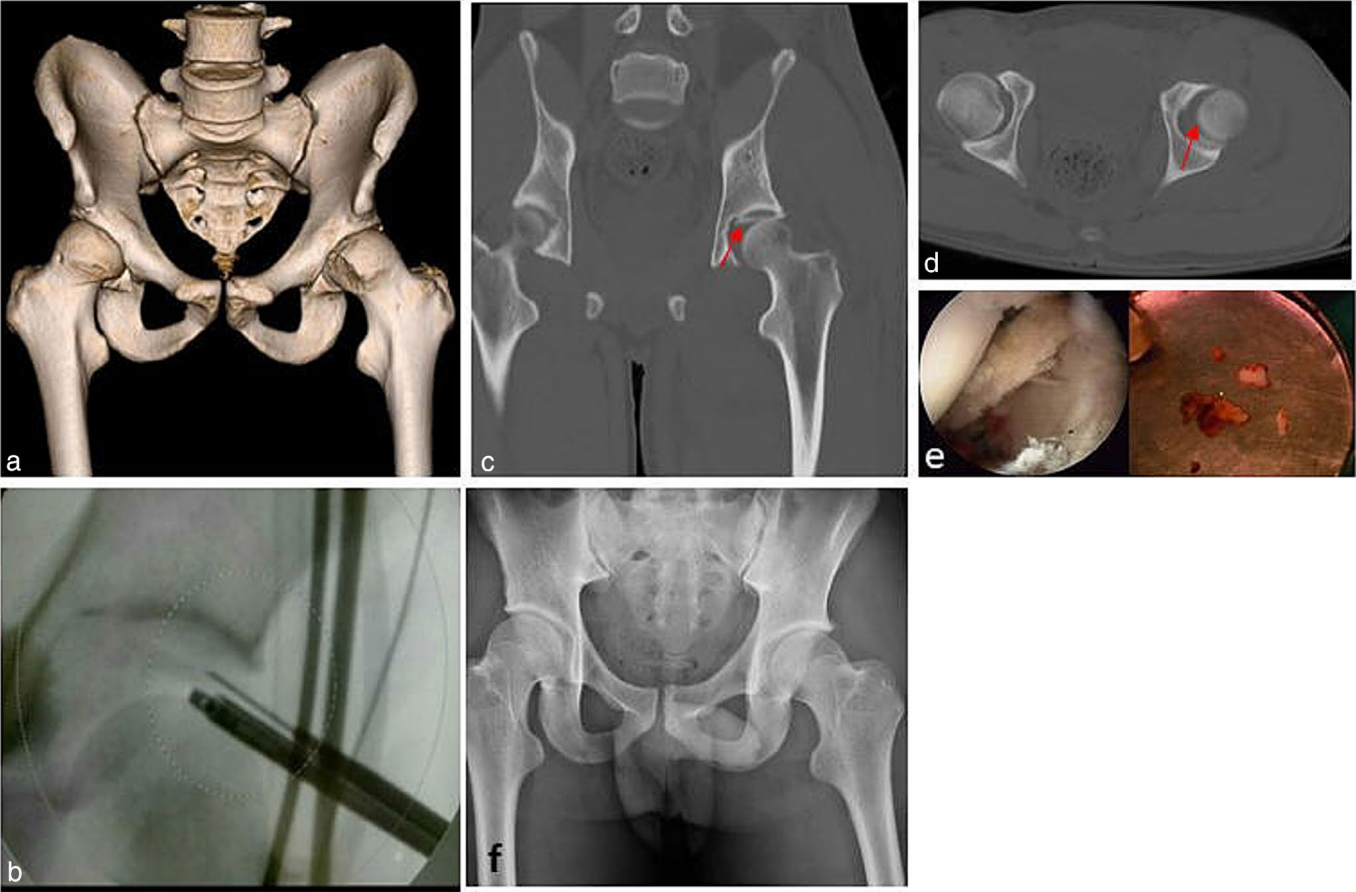
(a–f) A 19‐year‐old female with a left Pipkin Type I femoral head fracture–dislocation. Intraoperative arthroscopic views (e) showing removal of small osteochondral fragments and labral repair (arrow), and the result after 7‐year follow‐up (f).

The final reduction and hardware positioning were confirmed through direct arthroscopic visualization from multiple portals and dynamic fluoroscopic examinations. Any irreducible or small, nonstructural fragments were excised to prevent joint impingement (Figure 3). Concomitant lesions, such as labrum tears or loose osteochondral bodies, were addressed concurrently. No suction drains were utilized, and the portal wounds were closed primarily.

### 2.4 Postoperative Management

All patients received prophylactic first‐generation cephalosporin antibiotics for 24 h. Toe‐touch weight‐bearing with crutches was initiated on Postoperative Day 1. Clinical and radiographic follow‐up was conducted at 1, 3, 6, 12, and 24 months and subsequently at 5 and 10 years.

### 2.5 Outcome Evaluation

Clinical outcomes were evaluated using the Harris Hip Score (HHS) and Thompson–Epstein (T–E) criteria at the final follow‐up. Pain was assessed using the VAS within the first 24 postoperative hours. Radiographic outcomes included fracture reduction quality (Matta's criteria), residual displacement measured on 3D‐CT at 3 months for fixation cases, and the occurrence of AVN, Brooker classification of HO [10], and Tönnis classification of hip osteoarthritis (OA) [11].

### 2.6 Statistical Analysis

Continuous variables are presented as mean ± standard deviation. Categorical variables are presented as frequencies and percentages. Group comparisons were performed using independent‐samples *t*‐tests or Mann–Whitney *U* tests for continuous variables and Fisher's exact test for categorical variables due to the small sample sizes. Statistical significance was set at *p* < 0.05. Analyses were performed using SPSS Statistics 17.0 (IBM Corp., Armonk, NY, USA) and GraphPad Prism 8.0 (GraphPad Software, San Diego, CA, USA).

## 3 Results

The cohort of 23 patients (19 men and 4 women) constituted the final study reviewed, including 21 involved in motor vehicle accidents, one who sustained a fall from height, and one from a sports‐related injury. The mean time from initial injury to definitive surgical intervention was 3.6 ± 1.7 days (range, 2–9 days). All patients underwent emergency closed reduction of the hip dislocation in the emergency department immediately upon arrival. The interval between reduction and definitive surgery allowed for medical optimization and detailed preoperative planning using 3D‐CT imaging. The mean age at surgery was 28.7 ± 9.9 years, and the mean follow‐up duration was 86.2 ± 21.2 months. Nineteen patients sustained Pipkin Type I fractures and four sustained Pipkin Type II fractures. All patients underwent emergency closed reduction within 6 h of injury.

### 3.1 Clinical Outcomes

At final follow‐up, the mean HHS for the entire cohort was 98.3 ± 1.9. According to the T–E criteria, 21 patients (91%) achieved excellent outcomes, and 2 (9%) had good outcomes; no fair or poor results were observed.

Fifteen patients underwent arthroscopy‐assisted internal fixation and eight underwent fragment excision. There were no statistically significant differences between the fixation and excision groups with respect to HHS, postoperative pain scores, or operative time. However, the hospital stay was significantly shorter in the excision group compared with the fixation group (2.9 ± 0.6 vs. 5.5 ± 4.6 days, *p* = 0.028) (Table 1).

**TABLE 1:** Summary of patient demographics and fixation versus excision.

Variable	Overall (*n* = 23)	Fixation (*n* = 15)	Excision (*n* = 8)	*p* between fixation and excision
Age, years (mean ± SD)	28.7 ± 9.9	28.6 ± 10.1	28.9 ± 10.2	0.794
Sex, M/F	19/4	13/2	6/2	0.589
Pipkin Type I/II	19/4	11/4	8/0	0.257
Side of injury (right/left)	12/11	7/8	5/3	0.686
Mechanism (motor/falling/sports)	21/1/1	14/1/0	7/0/1	0.465
Surgical details				
Operative time, min (mean ± SD)	217.8 ± 49.8	219.6 ± 52.3	208.6 ± 45.3	1.000
Hospital stay, days (mean ± SD)	4.6 ± 3.9^a^	5.5 ± 4.6	2.9 ± 0.6	0.028*
Outcomes				
HHS at last follow‐up (mean ± SD)	98.3 ± 1.9	97.9 ± 1.8	99.0 ± 1.9	0.064
Thompson–Epstein (excellent/good)	21/2	13/2	8/0	0.515
VAS pain score, POD1 (mean ± SD)	3.4 ± 0.9	3.5 ± 1.0	3.3 ± 0.7	0.523
Complications	HO: 1 (4%), OA: 1 (4%), AVN: 0	HO: 0, OA: 1	HO: 1, OA: 0	

Abbreviations: HHS, Harris Hip Score; POD1, Postoperative Day 1; VAS, Visual Analog Scale.

^a^ Two patients with severe, life‐threatening polytrauma (e.g., traumatic brain injury, solid organ abdominal trauma) were excluded.

* *p* < 0.05.

Patients with Pipkin Type I fractures demonstrated slightly higher HHS than those with Pipkin Type II fractures (98.7 ± 1.7 vs. 96.0 ± 0.8, *p* = 0.018). Pipkin Type II fractures needed longer operative times than Pipkin Type I (291.7 ± 53.5 vs. 203.3 ± 36.5, *p* = 0.018) (Table 2).

**TABLE 2:** Comparison of Pipkin I and II groups.

Variable	Pipkin I (*n* = 19)	Pipkin II (*n* = 4)	*p*
Age, years (mean ± SD)	27.5 ± 8.8	34.2 ± 14.3	0.235
Sex, M/F	16/3	3/1	1
Operative time (min)	203.3 ± 36.5	291.7 ± 53.5	0.018*
Hospital stay (days)	4.7 ± 4.3	3.8 ± 0.5	0.344
VAS pain score (POD1)	3.4 ± 0.9	3.5 ± 1.0	0.92
HHS (last follow‐up)	98.7 ± 1.7	96.0 ± 0.8	0.018*
HO, *n* (%)	1 (5%)	0 (0%)	1
OA, *n* (%)	0 (0%)	1 (25%)	0.174

Abbreviations: AVN, avascular necrosis; HHS, Harris Hip Score; HO, heterotopic ossification; OA, osteoarthritis; POD1, Postoperative Day 1; VAS, Visual Analog Scale.

* *p* < 0.05.

### 3.2 Radiographic Outcomes

**FIGURE 4:**
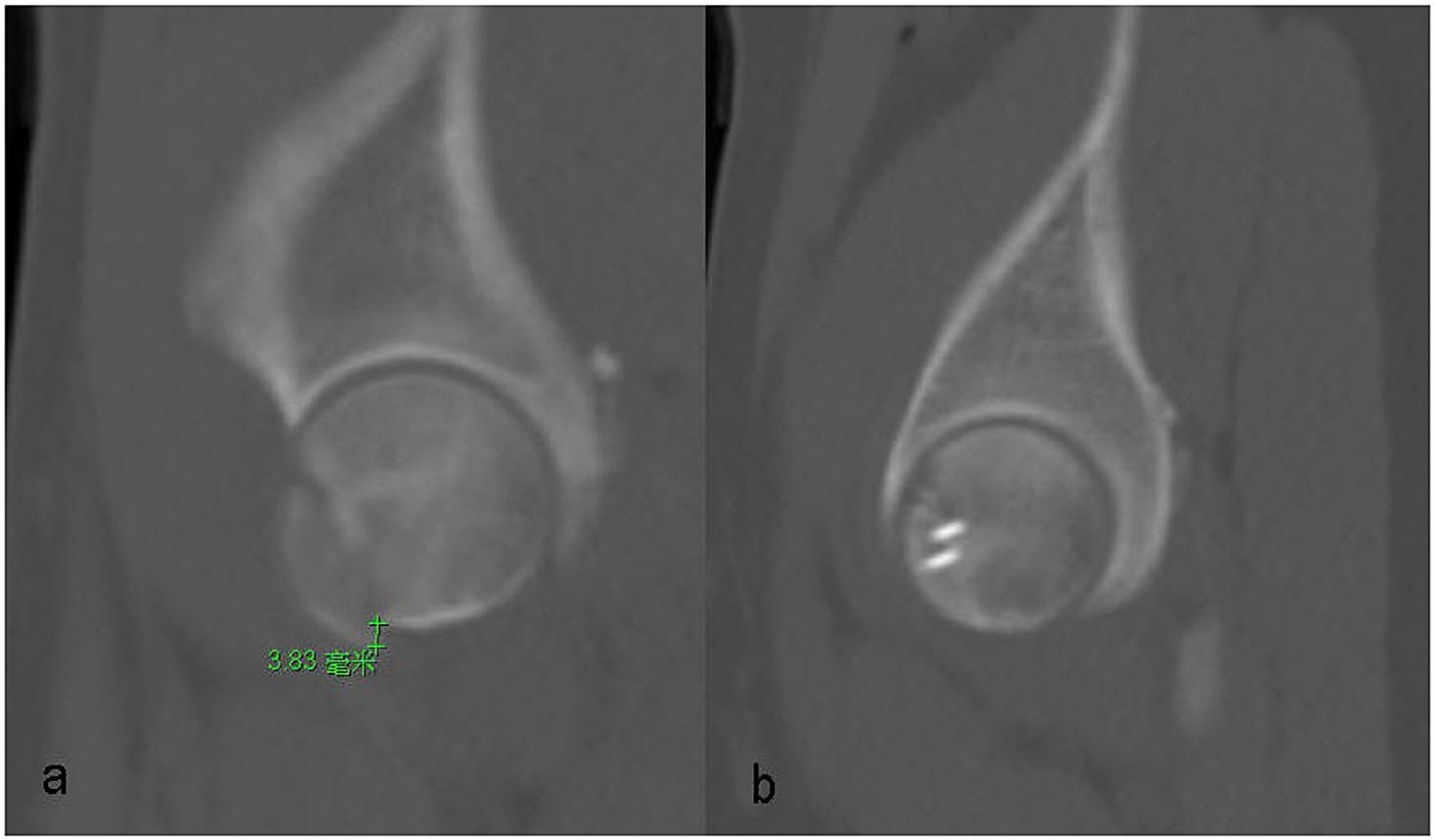
Measurement of maximal residual displacement on CT scans preoperatively (a) and postoperatively (b).

- *Fracture healing and time to union*: In all 15 patients treated with internal fixation, radiographic union was achieved, with evidence of bridging bone confirmed via CT scan at the 3‐month postoperative mark.
- *Reduction quality*: According to Matta's criteria, the postoperative reduction was classified as anatomic in six cases (40.0%), imperfect in six cases (40.0%), and poor in three cases (20.0%).
- *Displacement correction*: The mean maximal fracture displacement improved significantly from 7.6 mm preoperatively to 2.6 mm postoperatively (Figure 4).
- *Implant and fragment stability*: Throughout the minimum 5‐year follow‐up period, there were no observed instances of screw migration, implant failure, or secondary loss of reduction.
- *Bone resorption*: Importantly, no cases of partial or complete bony resorption of the fracture fragments were identified on follow‐up imaging.

### 3.3 Perioperative Parameters

The mean operative time in all cohorts was 218 min. On Postoperative Day 1, the mean VAS pain score was 3.4, and the mean length of hospital stay was 4.6 days. Patients with severe, life‐threatening polytrauma (e.g., traumatic brain injury, solid organ abdominal trauma) were excluded, as these conditions would independently dictate a prolonged hospital stay. Intraoperative blood loss was minimal in all cases, and no patient required a blood transfusion.

### 3.4 Complications

**FIGURE 5:**
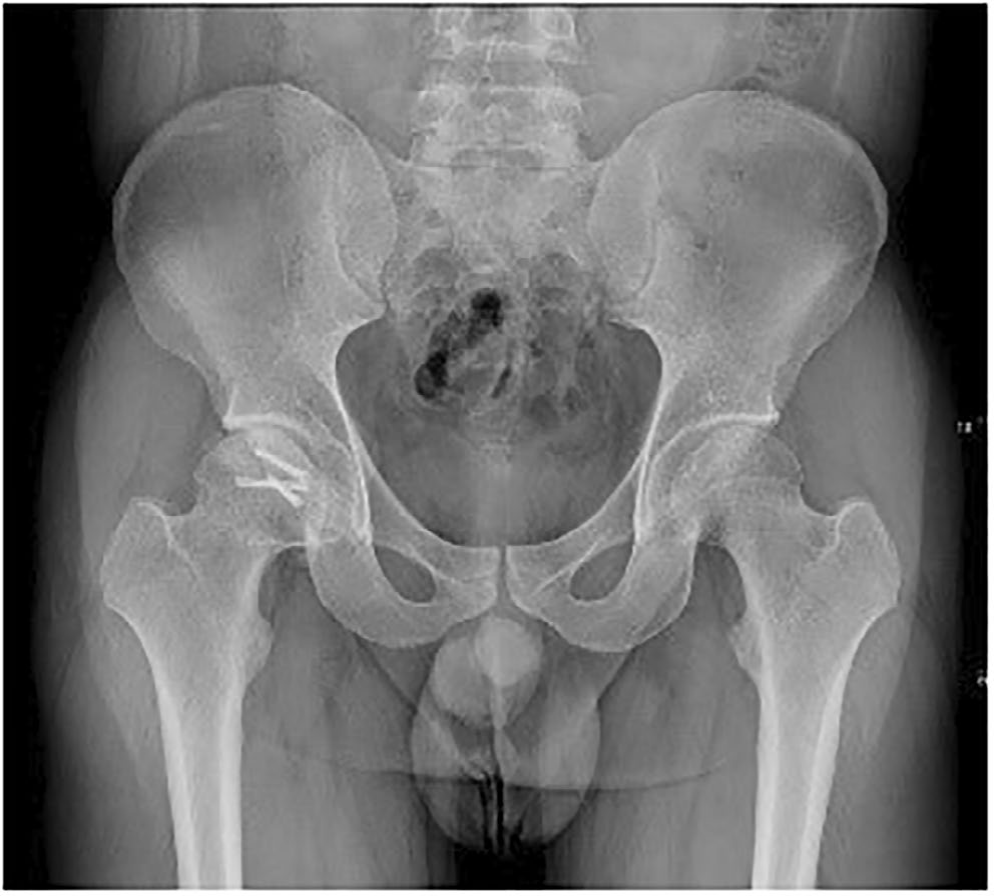
A 24‐year‐old male with a Pipkin Type II fracture at 9 years of follow‐up, showing stable fixation and mild osteoarthritis.

One patient in the excision group developed Brooker Grade I HO (4%), and one patient in the fixation group developed Tönnis Grade I OA (4%) at 9 years of follow‐up (Figure 5). One patient with an associated femoral shaft fracture experienced transient sciatic nerve palsy, which resolved completely within 3 months. No cases of AVN, deep infection, severe post‐traumatic arthritis, or conversion to total hip arthroplasty were observed during the follow‐up period.

## 4 Discussion

In the main findings, our study demonstrates that hip arthroscopy–assisted management of Pipkin Type I and II femoral head fractures yields excellent mid‐term clinical outcomes with a high rate of joint preservation. At a mean follow‐up of over 7 years, patients achieved a mean HHS of 98.3, with minimal complications and a 0% conversion rate to total hip arthroplasty. While Pipkin Type II fractures were technically more demanding, arthroscopic techniques provided a viable minimally invasive alternative to traditional open surgery.

### 4.1 Functional Outcomes and Surgical Approaches

Functional outcomes following femoral head fractures have historically been variable, with earlier studies reporting good or excellent results in only 40%–80% of cases, regardless of surgical approach [12]. Systematic reviews have demonstrated no clear superiority between anterior and posterior open approaches, with approximately two‐thirds of patients achieving acceptable outcomes based on T–E criteria [13]. Surgical hip dislocation through trochanteric flip osteotomy has reported a mean HHS ranging from 82 to 84.4, reflecting reliable exposure but substantial surgical morbidity [14, 15, 16]. Advances in surgical techniques and instrumentation have contributed to improved outcomes with less invasive approaches. Sun et al. reported a mean HHS of 95.9 following fixation through a direct anterior approach [17], while Kloub et al. achieved a mean HHS of 98.1 using minimally invasive osteosynthesis after successful anatomic reduction [18]. These findings suggest that satisfactory outcomes can be achieved using different strategies when appropriate fracture patterns are carefully selected. In this context, hip arthroscopy has emerged as a minimally invasive alternative for managing selected femoral head fractures. The outcomes are comparable to, or better than, those reported for open or surgical dislocation techniques in appropriately selected cases.

### 4.2 Complications and Safety Profile

Complication rates after femoral head fracture management vary with surgical approach [4, 19, 20]. Posterior approaches are associated with an increased risk of AVN due to potential disruption of the deep branch of the medial femoral circumflex artery, whereas anterior approaches have been linked to higher rates of HO [21]. A systematic review found that anterior approaches produced more HO (42%) but fewer AVN cases than posterior approaches [22]. Large pooled analyses have reported AVN rates of approximately 12%, post‐traumatic arthritis rates of 20%, and HO rates approaching 17% [23]. Surgical hip dislocation series report HO rates of 15%–33% and AVN rates of 8%–17% [14, 15, 16]. In contrast, our cohort demonstrated a low complication profile, with no cases of AVN or conversion to total hip arthroplasty and only one case each of mild HO and early OA at nearly 9 years of follow‐up. This favorable profile may be attributable to limited soft‐tissue disruption, preservation of femoral head vascularity, and careful patient selection inherent to arthroscopic techniques. Nevertheless, arthroscopy carries unique risks, including traction‐related neuropraxia, fluid extravasation, and iatrogenic cartilage injury, which necessitate meticulous technique and experience [24, 25].

### 4.3 Excision vs. Fixation and Fracture Subtypes

Although the excision group demonstrated a significantly shorter hospital stay, functional recovery was comparable between the two groups. This suggests that excision of small, nonstructural fragments is a highly efficient procedure, while fixation of larger fragments successfully preserves the femoral head's structural integrity without increasing mid‐term morbidity [9, 26]. Although patients with Pipkin Type II fractures required longer operative times and resulted in slightly lower final functional scores than Type I fractures. This likely reflects the technical difficulty of manipulating fragments located superior to the fovea capitis within the constrained intra‐articular space. However, both groups achieved excellent results, indicating that the minimally invasive nature of arthroscopy is beneficial regardless of the fracture subtype. Nevertheless, our results align with prior studies indicating that Pipkin Type I and II fractures can achieve comparable functional recovery following timely and appropriately selected surgical intervention [1, 2, 19].

Our final aim was to assess the impact of reduction quality on mid‐term clinical outcomes and joint preservation. In our fixation cohort, anatomic reduction was achieved in 40% of cases, while the remainder demonstrated imperfect or poor reduction according to Matta's criteria. Despite this, excellent functional outcomes were observed across the cohort, and no clear association between reduction quality and functional outcomes was identified at the mid‐term follow‐up. This finding may reflect the limited sensitivity of traditional radiographic criteria in capturing subtle cartilage or labral congruity, particularly in arthroscopic procedures where direct visualization allows targeted management of intra‐articular pathology. In addition, small residual step‐offs may be clinically tolerated in selected infrafoveal fractures without compromising mid‐term function. However, the relatively low rate of anatomic reduction highlights a technical limitation of arthroscopic fixation, particularly with respect to rotational control, and underscores the importance of surgeon experience and careful case selection.

### 4.4 Surgical Tips

- *Vascular mapping*: Utilizing preoperative ultrasonography to identify the femoral artery is a critical safety step before establishing the anterior portals to prevent iatrogenic vascular injury.
- *Positional advantage*: Placing the hip in maximal external rotation is the key maneuver to bring the fracture fragment superiorly into the optimal operative visual field.
- *Reduction technique*: A “joystick technique” using standard arthroscopic graspers or elevators is highly effective for manipulating fragments within the constrained intra‐articular space.
- *Safe trajectory for fixation*: When inserting percutaneous guide wires, strictly constrain the entry point between the anterior arthroscopic portal and the ASIS. Employ blunt muscle dissection to protect the surrounding soft‐tissue envelope.
- *Hardware management*: To prevent devastating iatrogenic cartilage wear, it is imperative to meticulously countersink the 2.0‐mm Herbert screws beneath the subchondral bone. Dynamic fluoroscopy should be used to confirm that the screws do not protrude during any range of motion.
- *Impingement prevention*: Surgeons should maintain a low threshold for excising small, comminuted, or irreducible nonstructural fragments. Forcing fixation on these pieces increases operative time and the risk of postoperative joint impingement.

### 4.5 Limitations and Strengths

The primary strength of this study is its substantial follow‐up duration for hip arthroscopy–assisted surgery of Pipkin Type I and II fractures, which is long enough to definitively capture late‐onset complications. Second, this approach demonstrated high functional outcomes and an excellent safety profile by preserving the vascular supply to the femoral head compared to traditional open procedures. Third, it provides an actionable clinical subgroup comparison between fragment excision and internal fixation. However, several limitations of this study should be acknowledged. The retrospective design, small sample size, and imbalance between subgroups limit statistical power. Selection bias is inherent, as arthroscopy was reserved for fractures deemed technically suitable, and two patients required intraoperative conversion to open surgery. Furthermore, anatomic reduction was achieved in only 40% of fixation cases, compared with surgical dislocation techniques, which report higher rates of perfect reduction [15, 16]. The relatively long operative time also highlights the steep learning curve associated with this technique. Finally, the absence of a contemporaneous open‐surgery control group limited the direct comparison of outcomes and complication rates.

## 5 Conclusions

Hip arthroscopy–assisted management of selected Pipkin Type I and II femoral head fracture–dislocations provides excellent mid‐term functional outcomes with a low incidence of complications. This minimally invasive technique represents a viable alternative in appropriately selected patients when fragment characteristics and surgical expertise permit.

## Author Contributions

**Chi‐Hsiang Hsu:** methodology, resources, data curation. **Shan‐Ling Hsu:** conceptualization, methodology, data curation, writing – original draft, writing – review and editing, resources. **Chin‐Yi Liao:** data curation, formal analysis.

## Funding

The authors have nothing to report.

## Ethics Statement

This study was approved by the Institutional Review Board of Chang Gung Medical Foundation (Date 2017.06.7/No. 201700816B0). Data were collected retrospectively from the institutional registry, and all patients provided written consent for their data to be used in this study.

## Conflicts of Interest

The authors declare no conflicts of interest.

## Data Availability

The data that support the findings of this study are available on request from the corresponding author. The data are not publicly available due to privacy or ethical restrictions.
